# Cell Hydration as a Biomarker for Estimation of Biological Effects of Nonionizing Radiation on Cells and Organisms

**DOI:** 10.1155/2014/890518

**Published:** 2014-12-18

**Authors:** Sinerik Ayrapetyan, Jaysankar De

**Affiliations:** UNESCO Chair in Life Sciences International Postgraduate Educational Center, 31 Acharian Street, 0040 Yerevan, Armenia

## Abstract

“Changes in cell hydration” have been hypothesized as an input signal for intracellular metabolic cascade responsible for biological effects of nonionizing radiation (NIR). To test this hypothesis a comparative study on the impacts of different temperature and NIR (infrasound frequency mechanical vibration (MV), static magnetic field (SMF), extremely low frequency electromagnetic field (ELF EMF), and microwave (MW)) pretreated water on the hydration of barley seeds in its dormant and germination periods was performed. In dormant state temperature sensitivity (*Q*_10_) of seed hydration in distilled water (DW) was less than 2, and it was nonsensitive to NIR treated DW, whereas during the germination period (48–72 hours) seeds hydration exhibited temperature sensitivity *Q*_10_ > 2 and higher sensitivity to NIR treated DW. Obtained data allow us to suggest that the metabolic driving of intracellular water dynamics accompanied by hydrogen bonding and breaking is more sensitive to NIR-induced water structure changes in seed bathing aqua medium than the simple thermodynamic processes such as osmotic gradient driven water absorption by seeds in dormant state. Therefore, cell hydration is suggested to be a universal and extrasensitive biomarker for detection of biological effects of NIR on cells and organisms.

## 1. Introduction

Although the biological effects of NIR including MV, SMF, ELF EMF, and MW on cells and organisms are considered as proven facts, the nature of cellular target(s) through which their biological effects are realized is still unclear [1–3]. This acts as the main barrier in comprehending the cellular and molecular mechanisms of biological effects of NIR. This also hinders deducing an adequate dosimetry for NIR from the point of public health and ecosystem management. There are a number of hypotheses in this regard but none of them is able to reliably explain the great number of experimental data on biological effects of NIR obtained in different laboratories. The difficulty lies in the fact that the biological effects of any weak environmental signal depend not only on its thermodynamic characteristics but also on its frequency, chemical and physical composition of the surrounding medium, and the initial metabolic state of the organisms. However, the World Health Organization (WHO), International Commission of Non-Ionizing Radiation Protection (ICNIRP), and other organizations, having the mission and role of monitoring hazardous effects of NIR, traditionally base their instructions only on the quantity of NIR energy absorption (specific absorption rate (SAR)) by cells and organisms.

As the biological effects of NIR on organisms could be recorded also at their intensities much less than thermal threshold [4, 5] these effects cannot be explained by the hypothesis based on classic thermodynamic characteristics of NIR [6]. Therefore, some researchers try to explain these effects from the point of quantum mechanical approach considering the processes in which the transfer of uncoupled electrons, such as the electron transferring from Cytochrome C to Cytochrome oxidase, oxidation of malonic acid, Na^+^/K^+^-ATP-ase, and others, take place [7–10]. However, it is suggested that the extrahigh NIR sensitivity of valence angle in water molecule from one side and high permeability of cell membrane for water from the other side make the NIR-induced changes of cell hydration a gate of metabolic cascades through which its biological effects are realized. It is known, that the cell hydration-induced changes of cell metabolic activity could be realized by the following 3 pathways: (a) by changes of hydration (folding-unfolding) of intracellular micromolecules determining their activity [11], (b) by modulation of collective dynamics of intracellular water in living cells accompanied by the processes of breaking and forming of hydrogen bonds [12, 13], and (c) by cell surface-dependent changes of a number of functionally active proteins in membrane [14, 15].

The plant seeds, being in dormant (metabolically inactive) and germination (metabolically active) states, serve as an ideal experimental model for checking this hypothesis. It is known that plants germination and growth are NIR-sensitive biological processes [16–26].

Usually four stages of organogenesis of seed growth can be distinguished at room temperature: 1st stage (seed swelling until critical moisture and active functioning of the enzymes are achieved) at 2 hours, 2nd stage (awakening) at 24 hours, 3rd stage (germinal root formation) at 48 hours, and 4th stage (germination) at 72 hours [27]. As the effects of temperature on rates of diffusion and chemical reaction (metabolic) are well described in thermodynamics, it was suggested that the study of temperature sensitivity of water absorption by plant seeds could be used to distinguish between the gradient (osmotic) driven and metabolic controlling of the seeds water absorption at different incubation periods in aqua medium. In present study the barley seed germination* in vitro* was used as an experimental model. The time-dependent (2, 24, 48 and 72 hours) water absorption by dry seeds incubated in Sham and NIR treated DW in cold (4°C) and warm (20°C) conditions was studied.

## 2. Materials and Methods

### 2.1. The Preparation of Water and Seed Samples

Grains of spring barley (sort-Nutans 115, forming fibrous root systems, cleistogamous) cultivated in the Shirak valley (Armenia) were supplied by the Echmiadzin Research Center of Agriculture and Plant Protection (Armenia) for experimental purpose.

DW was used as the seed incubation medium having an initial conductivity in the range of 1–10 *μ*s/cm at room temperature (20°C). It was poured into clean sterilized glass container. The DW was kept frozen for 24 hours and then thawed at room temperature and an hour later it was used for seed incubation in all experiments.

**Figure 1:**
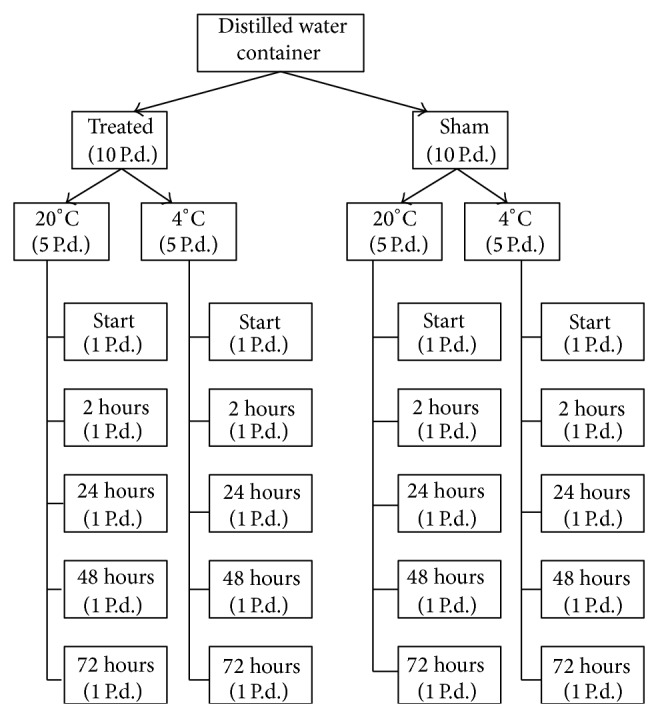
Protocol of experiments of the study of Sham and NIR treated DW effects on seed hydration at the end of 2, 24, 48, and 72 hours of incubation in DW.

For the DW treatment the following NIR sources were used: MV—10 Hz, 30 dB, SMF—2.5 mT, ELF EMF—15 Hz, 2.5 mT, and MW-SAR = 1.96 mW/kg. NIR and Sham treatment periods were 15 minutes. The Sham treated DW was put in the same setups for 15 minutes but in their “switch-off” states (control). The NIR-treatments of DW and their effects on seed hydration were performed by the methods described earlier [17]. The schema of the experimental protocol for elucidating each NIR (MV, SMF, ELF EMF, and MW) effect on cell hydration of seeds is presented in Figure 1.

1 liter of DW was divided into 500 mL for NIR (MV, SMF, ELF EMF, and MW) and 500 mL for Sham exposure. After NIR and Sham exposures each 500 mL DW was added to 10 Petri dishes (Pd), respectively; thus, each Petri dish contained 50 mL of DW and 100 seeds. Both NIR and Sham treated dishes containing DW and seeds were divided into 2 sets, respectively. The first columns of both NIR and Sham treated dishes were incubated at 20°C (5 Pd in each column), while the second columns of NIR and Sham treated dishes were incubated at 4°C (5 Pd) in each column. The time-dependent changes (0, 2, 24, 48, and 72 hours) of seed hydration were measured in Sham and NIR treated samples (1 dish, 100 seeds) at 4°C and 20°C, respectively.

This moment was considered as the starting time of seed incubation, which lasted for 72 hours at 4 or 20°C depending on the protocol (0, 2, 24, 48, and 72 hours of incubation) (Figure 1). At the beginning of the experiment all seeds were weighed (wet weight, w.w.). In each experiment the initial weight of the seeds varied in the range of 40–50 mg per seed. The value of seed dry weight was obtained by drying one set of them at 104°C for 24 hours in thermostat [22] and was sacrificed at predetermined time intervals. Analyses for initial dry weight and hydration were done after 5 minutes from this starting point. To exclude the effect of light all the experiments were performed in the dark and the experiments were run under “blind” condition; that is, the experimenter did not know the treatment conditions of the seed samples.

By following determination of initial w.w. the experimental sets of seeds were incubated in Sham and NIR treated DW for 2, 24, 48, and 72 hours. Thus, we had the opportunity to calculate the water content in the seeds at different periods of their growth. Seed hydration (mg water/mg dry seed) was determined by (w.w.−d.w.)/d.w. Measurement of the weight of seeds was done by precise analytical balance.

This setup of experiments for each NIR effect on seed hydration was repeated for 3 times with 3 days of interval. As a result each sample contained 300 seeds (3 dishes).

### 2.2. Experimental Setup and Sample Treatment

**Figure 2:**
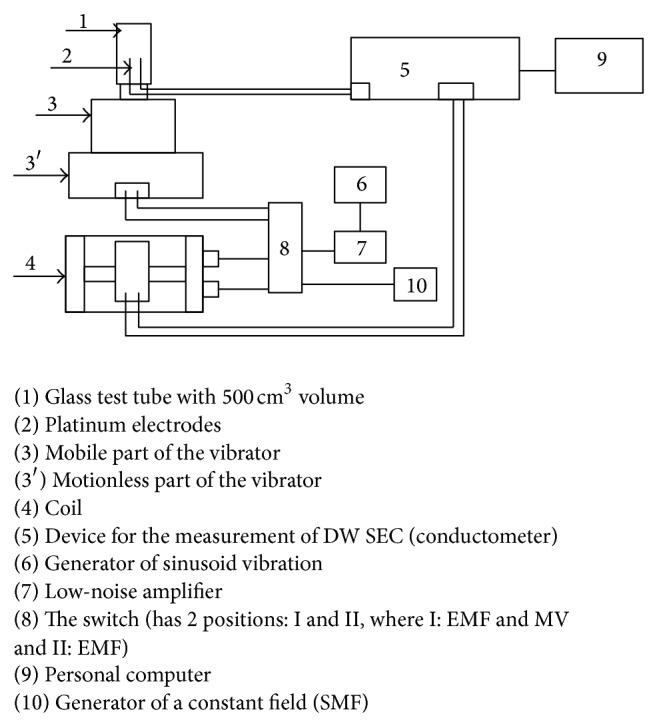
Setup for treatment of DW by NIR: (1) glass test tube with 500 cm^3^ 10 mL; (2) platinum electrodes; (3) mobile part of the vibrator; (3′) motionless part of the vibrator; (4) coil; (5) device for the measurement of DW SEC (conductometer); (6) generator of sinusoid vibration; (7) low-noise amplifier; (8) the switch (has 2 positions: I and II, I: EMF and MV and II: EMF); (9) personal computer; (10) generator of a constant field (SMF).

The block schema of setup for treatment of DW by ELF EMF, SMF, and MV is presented in Figure 2. This setup was assembled at the Institute of Radiophysics and Electronics (IRPHE) of Armenian National Academy of Sciences, Yerevan, Armenia, allowing the treatment of DW by MV, SMF, and ELF EMF. A block schema of this setup consisted of glass test tube with 500 cm^3^ volume, Helmholtz coils, low-noise amplifier, and a generator for generation of a homogeneous field as its main components. The vibrator was controlled by a sine wave generator (6) (GZ-118, made in Russian Federation) and the signal directed to the double pole switch (8). At the switch position I the generator functioned as EMF and MV sources, whereas at position II, it functioned as MV sources. The MV waves were obtained from the vibrating device (3) as it was used for generating vertical vibrations by setting frequency and intensity. A coil (4) with a feedback amplifier system (IRPHE, Yerevan, Armenia) was used to keep vibration intensity constant (30 dB) at different frequencies. The frequency of MV was controlled by a cymometer (CZ-47D, production of Russian Federation). The SMF was obtained by the generation of a static field (10) that was transferred through the coil (Figure 2).

The controlled generator and low-noise amplifier on a coil were used to generate the ELF EMF, the frequency of which was measured. The Helmholtz coil had a cylindrical form with a dimension of 154 mm in diameter and 106 mm in height. The coil consisted of Helmholtz rings generating a homogeneous magnetic field. Rings of Helmholtz were formed by two equal ring coils located coaxially and parallel. The distance between the coils was equal to their radius (77 mm). The magnetic field created by these rings had high homogeneity. For example, at a distance of 0.25 cm from the center of the axis, the strength differed by only 0.5% from the value computed by formula *H* = 71.6∗*ω*∗*I*/*R*, where *H* was magnetic field,  *ω* was density, *I* was amperage, and *R* was resistance. SMF was generated by the generator of a static field (10) and transferred to the coil.

The intensity of the magnetic field was measured by a Hall detector (sensitivity 265 V/mT). Frequency of ELF EMF was measured by frequency meter CП-4 (Russian production, Nowosibirsk). Based on our previous finding that the 15 Hz (2.5 mT) ELF EMF is the most effective frequency window, having activation effect on barley seeds germination [17] in our current experiments, this frequency for ELF EMF treatment of DW was used.

### 2.3. MW Radiation Source

“Artsakh-04M” device (Quantum Medicine Association Coloyaro-2000, Moscow, Russian Federation) was used as MW source for MW therapy. It consisted of a microprocessor managing system for solid-state generation of MW in the ranges of 90–160 GHz with amplitude modulation at 0–99 Hz pulse-rate. As it was previously shown that 4 Hz-modulated MW has more pronounced effect on water structure [28] this modulation was used in the present experiment. The needle-type of dielectric antenna was inserted in cup containing DW for MW treatment.

### 2.4. Determination of SAR for DW

The MW-SAR for DW was estimated by the following method: SAR of 1 mL DW was measured by differential calorimetric device “Biophys MWD-001” of high precision [29]. The main working principle of the device was based on electronic leveling of MW-induced temperature increase of the studying object (DW) and the temperature increase by alternating low frequency current on the compared object. The energy releasing from the antenna was measured by inserting its tip into DW. It was suggested that because of high absorption of DW all energy releasing from the tip would be fully absorbed by DW. SAR for DW was estimated by the following equation: SAR = CDWΔ*T*/Δ*t*, where CDW is DW specific heating capacity, Δ*T* is the change of absolute temperature, and Δ*t* is change of time.

### 2.5. Statistical Analysis

Each experimental sample (version) consisted of three dishes, each containing 100 seeds (total 300 seeds). For statistical validity, each stage of the experiment was repeated 3 times. A multifactor ANOVA to test for main and interaction effects followed by a multiple range test to compare two treatment groups was used for data analysis. The average, standard deviations and confidence limit (ANOVA), and so forth were calculated using MS-Excel program. The ^∗^ *P* < 0.05 was considered statistically significant.

## 3. Result

### 3.1. Temperature Sensitivity of Seed Hydration

**Figure 3:**
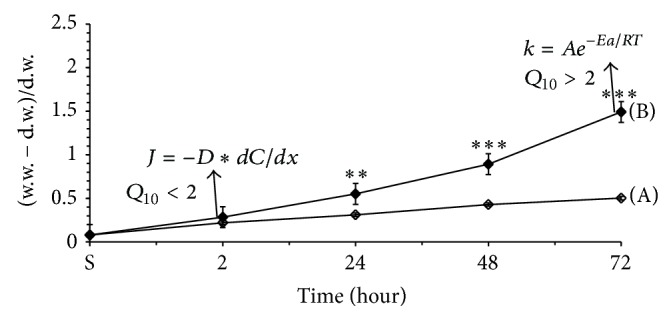
Time-dependent changes in seed hydration during 72 hours of incubation in nontreated (“*C*” = Sham) DW in cold (4°C; (A) clear symbol) and warm (20°C; (B) dark symbol) conditions. S = initial reading at 5 minutes after the start of the incubation. In the present and the following figures: “*C*” means Control-Sham. The time (in hours) of seeds incubation on abscissa and the value of seed hydration (mg of H_2_O for 1 mg of dry weight) on ordinate are presented. The results are shown as mean ± SEM (*n* = 300) from three independent experiments, 100 seeds from each experiment. ^∗^ *P* < 0.05, compared with Sham, ^∗∗^ *P* < 0.01.

The temperature sensitivity (*Q*_10_ coefficient) of the rate of processes serves as a marker for detecting whether they have diffusion or metabolic nature; that is, they are determined by Fick's law (*Q*_10_ < 2) and Arrhenius's equation (*Q*_10_ > 2), respectively. The time-dependent temperature sensitivity of seeds hydration incubated in DW in cold (4°C) and at room (20°C) temperature is shown in Figure 3.

The consequential changes in wet weights during seed incubation in control and experimental medium were expressed in percentage of their initial value before incubation. It can be seen that during incubation at both temperatures (4° and 20°C) there were increases in water absorption by seeds (2, 24, 48, and 72 hours of incubation). However, in the cold medium the rate of water absorption was much less than in the warm one and these differences had time-dependent increasing character. At the end of 2 hours of incubation, when seeds were in dormant state, *Q*_10_ was 1.25 for water absorption, while at the end of 72 hours of incubation at room temperature, when seeds were in germination state, the *Q*_10_ was 2.13. From these data it can be concluded that unlike dormant state, in germination state water absorption by seeds has a metabolic controlling character.

In order to find out whether NIR-induced water structure changes have impacts on metabolic controlling of seed hydration in next series of experiments the effects of different NIR (MV, SMF, ELF EMF, and MW) treated DW on seed hydration in their metabolically active and dormant states were studied.

### 3.2. Effect of MV-Pretreatment of DW on Seed Hydration

**Figure 4:**
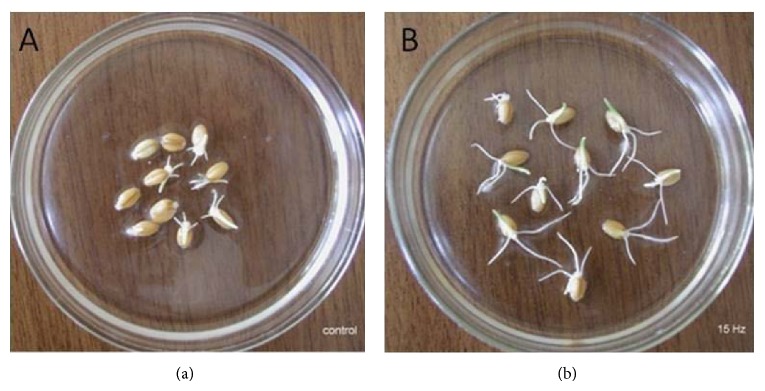
Barley seeds germination at the end of 72 hours, which were 15 minutes preincubated in Sham (control) (a) and 15 Hz MV treated (b) DW.

We have previously shown that 15 Hz MV pretreated DW has activation effect on germination potential of barley seed [17]. In Figure 4 it is shown that the seeds germination at the end of 72-hour incubation in 15 Hz MV treated warm DW water was significantly higher than in nontreated (Sham) warm DW water.

**Figure 5:**
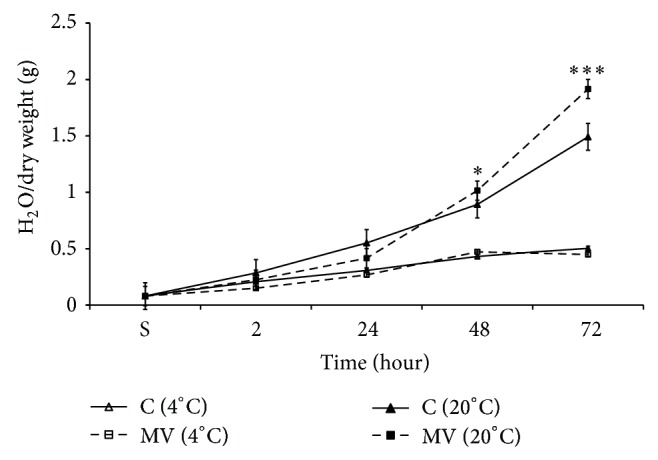
Time-dependent changes in seed hydration during 72 hours of incubation in MV (15 Hz; 30 dB) treated (gray column) and nontreated Sham (clear column) DW in cold (4°C; clear/grey with no pattern markings) and warm (20°C; clear/grey with horizontal pattern markings) conditions. The time (in hours) of seeds incubation on abscissa and the value of seed hydration (mg of H_2_O for 1 mg of dry weight) on ordinate are presented. The results are shown as mean ± SEM (*n* = 300) from three independent experiments, 100 seeds from each experiment. ^∗^ *P* < 0.05, compared with Sham, ^∗∗^ *P* < 0.01.

The time-dependent dynamics of seeds hydration changes during 72-hour incubation in Sham and 15 Hz MV treated warm DW showed that there were also significant differences between kinetics of their hydration (Figure 5).

The seed hydration was always less in MV treated DW than in the Sham until 24 hours of incubation. During the period of 48–72 hours of seed incubation (period of germination) clear increase in seed hydration rate in MV treated DW was seen compared to Sham treated DW. The *Q*_10_ for water absorption in cold DW was 0.97, whereas the same at room temperature was 2.33.

### 3.3. Effect of SMF-Pretreatment of DW on Seed Hydration

**Figure 6:**
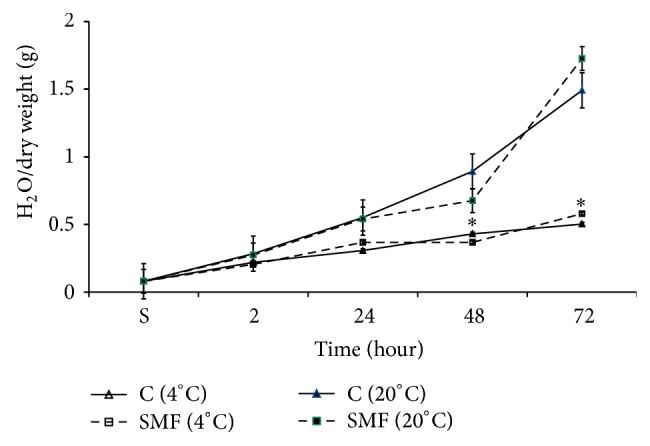
Time-dependent changes in seed hydration during 72 hours of incubation in SMF (2.5 mT) treated (gray column) and nontreated Sham (clear column) DW in cold (4°C; clear/grey with no pattern markings) and warm (20°C clear/grey with horizontal pattern markings) conditions. The time (in hours) of seeds incubation on abscissa and the value of seed hydration (mg of H_2_O for 1 mg of dry weight) on ordinate are presented. The results are shown as mean ± SEM (*n* = 300) from three independent experiments, 100 seeds from each experiment. ^∗^ *P* < 0.05, compared with Sham, ^∗∗^ *P* < 0.01.

It was seen that 2.5 mT SMF treated DW had enhancing effect on barley seed germination. The study on time-dependent seed hydration at room temperature in Sham and SMF treated DW showed that the latter has inhibitory effect on the rate of seed hydration during the period of 24–48 hours of incubation, while in the following period (48–72 hours of incubation) its effect on the rate of seed hydration was reversed and showed pronounced activation effect compared to seed hydration in Sham treated DW (Figure 6).

It is interesting to note that MV treatment had depressing effect on seed hydration compared to Sham, which in nature was similar to SMF treatment, but this depressing effect in case of SMF lasted till the end of 48 hours of incubation. However, at the end of 72 hours of incubation both MV and SMF pretreated DW showed activation effect on seed hydration. The *Q*_10_ for water absorption in cold DW was 1.31, whereas the same at room temperature was 2.11.

### 3.4. Effect of ELF EMF-Pretreatment of DW on Seed Hydration

**Figure 7:**
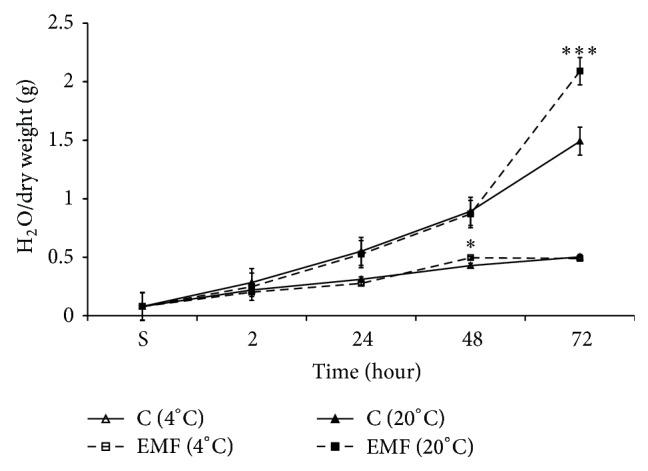
Time-dependent changes in seed hydration during 72 hours of incubation in ELF EMF (15 Hz) treated (gray column) and nontreated Sham (clear column) DW in cold (4°C; clear/grey with no pattern markings) and warm (20°C; clear/grey with horizontal pattern markings) conditions. The time (in hours) of seeds incubation on abscissa and the value of seed hydration (mg of H_2_O for 1 mg of dry weight) on ordinate are presented. The results are shown as mean ± SEM (*n* = 300) from three independent experiments, 100 seeds from each experiment. ^∗^ *P* < 0.05, compared with Sham, ^∗∗^ *P* < 0.01.

As can be seen in Figure 7 the 15 Hz ELF EMF treated DW had activation effect on seed hydration. Such activation effect was more expressed at the end of 72 hours of incubation at room temperature. It is worth to note that EMF sensitivity at room temperature was not expressed until the first 48 hours (so as at 4°C), whereas it was significantly higher at 72 hours of incubation. These data clearly indicate that the EMF-induced elevation of seed hydration was due to the metabolic processes corresponding to the time of incubation and those were activated in the course of germination. It is necessary to note that the latter can be considered as a significant increase in water content. The *Q*_10_ was 1.22 for water absorption in cold DW, whereas the same at room temperature was 2.70. These data showed that the metabolism-dependent seed hydration was more sensitive to EMF-induced water structure changes than the metabolism-independent one (in cold DW and during the first 2 hours of incubation in warm DW).

### 3.5. Effect of MW-Pretreatment of DW on Seed Hydration

**Figure 8:**
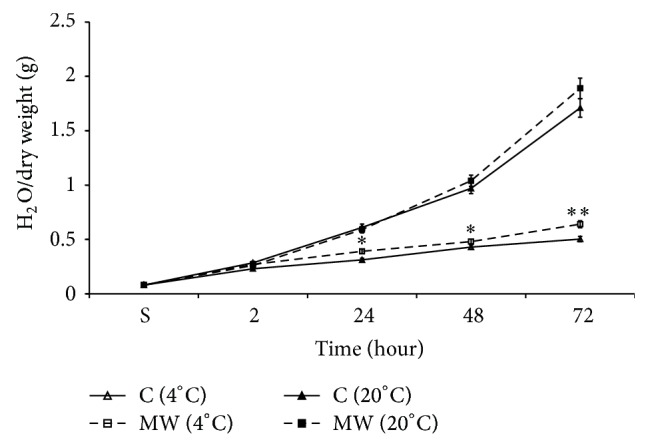
Time-dependent changes in seed hydration during 72 hours of incubation in MW treated (gray column) and nontreated Sham (clear column) DW in cold (4°C; clear/grey with no pattern markings) and warm (20°C; clear/grey with horizontal pattern markings) conditions. The time (in hours) of seeds incubation on abscissa and the value of seed hydration (mg of H_2_O for 1 mg of dry weight) on ordinate are presented. The results are shown as mean ± SEM (*n* = 300) from three independent experiments, 100 seeds from each experiment. ^∗^ *P* < 0.05, compared with Sham, ^∗∗^ *P* < 0.01.

In Figure 8 the data on time-dependence of seed hydration in Sham and MW treated DW in cold and at room temperatures is presented. It shows that the preliminary treatment with MW had a potentiating effect on seed hydration both in cold condition and at room temperature during the 72 hours of incubation. It is interesting to note that MW unlike ELF EMF (Figure 7) had elevation effect on seed hydration when compared with the Sham (Figure 8). The *Q*_10_ was 0.97 for water absorption in cold DW, whereas the same at room temperature was 2.08.

## 4. Discussion

The problem in elucidating the nature of target(s) for execution of biological effects of NIR, intensity of which is less than their thermal threshold, stays as one of the central problems of modern radiobiology. As the impact of such weak signals on biological process cannot be explained from the point of classic thermodynamics, it is suggested that the target(s) could have quantum mechanical nature [7, 9, 10]. However, the primary target(s) for biological effects of NIR stays discussable. One of the most popular hypotheses is the so-called “water” hypothesis according to which valence angle in water molecules between O–H bonds determining its physicochemical properties is highly sensitive to different environmental factors and serves as a primary target for NIR [30, 31].

High NIR sensitivity of water physicochemical (osmoactive) properties from one side and the cell membrane hyperpermeability for water molecules from the other side as well as metabolic driving of collective intracellular water dynamics allow us to suggest the metabolic controlling cell hydration as an extrasensitive biomarker of NIR effects. As the cell hydration determines its metabolic activity via “folding-unfolding” mechanisms of intracellular macromolecules [11] and cell surface-dependent changes of the number of functionally active protein molecules in plasma membrane [14, 15] its changing serves as a messenger for intracellular metabolic cascades through which the biological effects of NIR are realized. Therefore, the NIR-induced cell hydration changes could serve as a biomarker for estimation of NIR effects on cells.

Present experiment was dedicated to checking this hypothesis and sensitivity of seed hydration in its dormant and metabolic states to NIR-induced water structure changes. As indicated in Figure 3, the time-dependent water absorption in metabolically inactive states (first 2 hours of incubation in both temperatures and entire incubation period in cold medium) increased gradually, which can be considered as a passive osmotic gradient-driven water uptake ((A) in Figure 3). But in the germination period this water uptake depended on seeds metabolism (part (B) of Figure 3 after 2 hours of incubation in warm medium). This dependency was more pronounced in germination period from 48 to 72 hours.

Gradient-driven water absorption by dormant seeds must follow Fick's law for diffusion which is expressed as a function of osmotic active concentration gradient, /*dt* − *DA*(*dC*/*dx*), in which *M* means mass, *C* concentration, *D* diffusion coefficient (distance^2^/time), and *A* interfacial area (distance^2^). It is known that *D* is obtained from the equation *D* = *KRT*, where *K* is the coefficient for determining soluble substances, *R* is gas constant, and *T* is absolute temperature. From this equation the rate of diffusion is directly proportional to temperature, while metabolism-driven water absorption could be expressed as a rate of the lowest chemical reaction determining metabolic activity in seeds. Therefore, the metabolism-driven water absorption is temperature-dependent according to Arrhenius's equation, *k* = *Ae* ^−*E*_*a*_/*RT*^, where *k* is chemical reaction rate constant, *T* is absolute temperature, *R* is universal gas constant, and *E*_*a*_ is activation energy (reaction specific). From this equation, we can say that metabolism-driven water absorption must be more temperature-dependent than gradient-driven one. As from Van't Hoff's law the *Q*_10_, a factor by which the reaction rate increases when the temperature is raised by 10°C was far less than 2 in case of the osmotic absorption of water (dormant state of seeds), while in germination period it was more than 2, indicating the metabolic controlling of water absorption by seeds.

The data on insensitivity of seed hydration to NIR-induced water structure changes in dormant state (until the end of 48 hours of incubation at room temperature and in cold throughout 72 hours of incubation) and higher sensitivity in germination state (at the end of 72 hours of incubation at room temperature) clearly indicate that only metabolic-driving water adsorption by seeds is sensitive to water structure changing in seed bathing medium. It is known that during metabolic activity formation of new molecules of H_2_O, CO_2_ and other metabolites lead to activation of collective dynamics of intracellular water and continuous formation and breaking of hydrogen bonds, which are sensitive to environmental factors including NIR [13].

It is known that MV has elevation effect on plant germination [17, 22, 23]. It was previously shown that mechanical vibration at infrasound frequency leads to decrease in specific electrical conductivity (SEC) of water, which was due to decrease in CO_2_ solubility [32]. The obtained data from present work indicate that seed incubation in 15 Hz MV treated DW had more pronounced modulation effect on water absorption on seeds in germination period, which was less expressed in dormant state (Figure 5). From these data it can be concluded that water structure changes as a result of vibration of clustered water molecules due to strong modulation effect of MV treatment on water absorption by seeds in germination period.

There is a great number of literature data on SMF activation effect of plant seed germination [24–26]. It is known that SMF has depression effect on water dissociation by decreasing valence angle, which also leads to decrease of CO_2_ solubility in water and its SEC [30, 32]. It was previously shown that SMF treated solution also has activation effect on germination potential of seeds at 72 hours [17]. Therefore, the data presented in Figure 6 on SMF-induced increase in water absorption during germination period as in the case of MV treated solution can be explained by metabolic activity of seeds. As depicted in Figure 7 metabolism-dependent water absorption was more sensitive to structural changes in ELF EMF pretreated water too. The data presented in Figure 8 indicate that MW treated DW as in the case of ELF EMF also had elevation effect on seeds hydration at the end of 72 hours of incubation.

Our data have shown that MW has nonthermal effect on physicochemical properties of water and water solution [28] and literature data have also shown that MW with nonthermal intensity (mobile phone radiation) has also an activation effect on plant seed germination [21]. Therefore, these data can be considered as a strong evidence for nonthermal effect of MW on seed hydration which is realized through structural change in cell bathing aqua medium. As can be seen from the hydration pattern in case of EMF treatment (Figure 7) and MW treatment (Figure 8) at 48 hours they were opposite to each other. In case of the former it showed a depressing effect, whereas in case of the latter it was reversed. It indicates the fact that there is a possibility of differences in metabolic pathways occurring at these two different conditions even though they represented the same growth stage. It was predicted that the nonthermal effect of MW on water structure would predetermine the nonthermal biological effect of MW on different tissues and cells [33].

Thus, the obtained data in the present work bring us to the following conclusions. In dormant state water absorption of seeds has osmotic gradient driving character (*Q*_10_ < 2), while in germination state it has metabolic controlling character (*Q*_10_ > 2). Effects of NIR treated water on seed hydration in dormant and germination states are different from the effects of temperature (thermal). Thermal effect has activation effect on seed hydration throughout the whole incubation period, while NIR has effect only during germination period (48–72 hours) and, thus, this effect is nonthermal in nature.

The effects of NIR-induced water structure changes on seed hydration in germination period cannot be explained from the point of classic thermodynamics as its target has a quantum mechanical character.

The higher NIR sensitivity of water absorption in germination period can be explained by metabolic driving intracellular collective water dynamics, which is accompanied by breaking and formation of hydrogen bonds. Therefore, the cell hydration is suggested as a universal cellular marker for detection of biological effects of NIR.
